# Profile of scientific production on nursing technology construction, validity and application: a bibliometric study

**DOI:** 10.1590/0034-7167-2023-0452

**Published:** 2024-07-29

**Authors:** Fernando Conceição de Lima, Taís dos Passos Sagica, João Lucas Moraes Souza, Marta Lenise do Prado, Mary Elizabeth de Santana, Ivonete Vieira Pereira Peixoto, Rubenilson Caldas Valois

**Affiliations:** I Universidade do Estado do Pará. Belém, Pará, Brazil; II Universidade Federal de Santa Catarina. Florianópolis, Santa Catarina, Brazil

**Keywords:** Nursing Methodology Research, Nursing, Technology, Educational Technology, Bibliometric Analyses, Investigación Metodológica en Enfermería, Enfermería, Tecnología, Tecnología Educacional, Bibliometría

## Abstract

**Objective:**

to analyze the profile of scientific production on nursing technology construction, validity and application.

**Methods:**

this is a bibliometric study, carried out in six databases, based on the Methodi Ordinatio application, arranged in nine stages. To represent the findings, the VOSviewer^®^ software was used.

**Results:**

346 studies were identified, obtained from BDENF, CINAHL, EMBASE, LILACS, PubMed/MEDLINE, Scopus and Web of Science. There was a predominance of the English language, and 20% of the authors hold more than 25% of studies. Only two journals account for 25% of studies in the period studied. Twenty-six studies were selected for the InOrdinatio classification. Nursing Process (23%) stood out among the studies. The most produced technology was software (27%), and 50% of works describe construction and validity.

**Conclusions:**

there is an emphasis on the creation of educational technologies, especially information technology. The data demonstrates opportunities for future research in the area.

## RESUMEN

**Objetivo:**

analizar el perfil de la producción científica sobre la construcción, validación y aplicación de tecnologías en enfermería.

**Métodos:**

se trata de un estudio bibliométrico, realizado en seis bases de datos, basado en la aplicación del Methodi Ordinatio, dispuesto en nueve etapas. Para representar los hallazgos se utilizó el software VOSviewer^®^.

**Resultados:**

se identificaron 346 publicaciones, obtenidas de las bases de datos BDENF, CINAHL, EMBASE, LILACS, PubMed/MEDLINE, Scopus y Web of Science. Hubo predominio del idioma inglés y el 20% de los autores poseen más del 25% de las producciones. Sólo dos revistas son responsables del 25% de las publicaciones en el período estudiado. Se seleccionaron 26 publicaciones para la clasificación InOrdinatio. El destaque de las publicaciones fue el Proceso de Enfermería (23%). La tecnología más producida fue software (27%) y el 50% de los trabajos describen construcción y validación.

**Conclusiones:**

existe un énfasis en la creación de tecnologías educativas, especialmente tecnologías de la información. Los datos demuestran oportunidades para futuras investigaciones en el área.

## INTRODUCTION

The process of building technological devices permeates different formats and molds. Methodological research is the type that focuses on product development and usually involves complex methods. It is a methodology that can also be applied to investigations of the formats for obtaining and organizing data, in addition to conducting rigorous research^(1-2)^.

It is capable of handling research tool and method development, validity and assessment. In this type of study, the researcher aims to develop a reliable and usable product that can be used by other researchers and other people. This method is applicable in any scientific area, dealing with complex phenomena, such as individuals’ behavior or health^(1-2)^.

In nursing, there is a large number of methodological studies with a view to producing care, management and/or educational technologies. In addition to these, it is common to find proposals for international instrument assessment and cultural adaptation for use in the country of interest^(3-4)^. In general, the focus of methodological studies is on developing constructs applicable in the science and profession of nursing^(5)^.

As a main characteristic, these studies are presented in the form of chained, iterative and interactive processes, in which the stages of development of a technology rest. Some division models are currently assumed, such as carrying out situational diagnosis, organizing literature reviews, building technology, validating and testing^(3,6)^.

Broadly, the term “technology” can refer to techniques, methods, instruments, procedures and equipment that promote execution and generate products and services. In the health area, especially with regard to nursing service provision, the adoption of technologies is capable of refining professional practices, interpersonal relationships and the management of processes within services^(2,6)^. In this way, the application of technologies occurs in different ways and has its meaning attributed according to its use^(3)^.

Given the scientific visibility that methodological studies have gained and nursing engagement in the production of these studies, the question is how they are used by researchers in the development of new research^(7)^. Thus, carrying out this study proposes an analysis of scientific activity, through quantitative study, on the use of different stages in methodological research in nursing, since there is no consensus in the literature on the methodological process in creating, validating and applying nursing technologies as well as exploring methods and aspects that contribute to reflections on production and scientific advancement in this field^(7-8)^.

## OBJECTIVE

To analyze the bibliometric profile of scientific production on nursing technology construction, validity and application.

## METHODS

### Ethical aspects

The research was not submitted for consideration by a Research Ethics Committee, given that it was carried out with secondary data and in the public domain. However, study copyright was preserved.

### Study design, period and place

This is a bibliometric study about nursing technology construction, validity and application. This method is characterized by highlighting metrics, indicators, production and scientific dissemination of a given topic^(9)^.

It was based on *Methodi Ordinatio*, a multi-criteria aid tool that allows articles to be ordered considering three factors: number of citations; Impact Factor (metric); and year of publication^(8)^. Furthermore, the Preferred Reporting Items for Systematic Reviews and Meta-Analyses (PRISMA)^(10)^ flowchart guidelines were used, present in the study protocol registered on the Figshare platform^(11)^.

In this regard, this study followed the nine proposed steps: 1) research intention definition; 2) preliminary research in databases; 3) definition and combination of keywords and databases; 4) definitive search and data collection; 5) filtering procedure; 6) identification of Impact Factor, year of publication and number of citations; 7) classification of articles in *InOrdinatio* using the Rankln spreadsheet; 8) location of texts in full format; 9) systematic reading and analysis of articles^(12)^.

A preliminary search was carried out in the following databases: Nursing Database (BDENF); Cumulative Index to Nursing and Allied Health (CINAHL); EMBASE; Latin American and Caribbean Literature in Health Sciences (LILACS); MEDLINE via National Library of Medicine (PubMed); Scopus; and Web of Science (WoS). Data was collected from May to June 2023.

### Population or sample; inclusion or exclusion criteria

In the first stage, the PICo^(13)^ strategy was used to formulate the research question “What is the profile of scientific productions on nursing technology construction, validity and application?”, with P (problem): nursing technologies, I (interest): construction, validity and assessment studies, and Co (context): nursing in its multiple intervention scenarios.

Scientific articles on nursing technology construction, validity and/or application, available in Portuguese, Spanish and English, were included. The time frame from 2011 was used, as in that year there was an increase in studies (theses and dissertations) on the topic in the area, in addition to the expansion of graduate programs, especially professional programs^(8)^. Articles in editorial formats, letters to the editor and opinion articles were excluded.

### Study protocol

After the second stage of preliminary research in the databases, the search for studies began through remote access to the Federated Academic Community (CAFe - *Comunidade Acadêmica Federada*) content, a resource made available by the Coordination for the Improvement of Higher Education Personnel (CAPES - *Coordenação de Aperfeiçoamento de Pessoal de Nível Superior*) and the Ministry of Education (ME), signed by the *Universidade do Estado do Pará* (UEPA), through the combination of Descriptors in Health Sciences (DeCS)/Medical Subject Headings (MeSH)/Emtree (linked to the Embase Index), with the help of the Boolean operators AND and OR.

In the third stage, to define the keywords, Portuguese and English were used with the following combination: (“*pesquisa metodológica em enfermagem*” OR “nursing methodology research”) AND (“*enfermagem*” OR “nursing”) AND (“*tecnologia educacional*” OR “educational technology” OR “*tecnologia*” OR “technology” OR “*tecnologias*” OR “technologies”). This search strategy was standardized in databases and electronic portals, as shown in Chart 1.

**Chart 1:** Search strategy in databases and electronic portals, 2023

Database	Search strategy
BDENF/ LILACS	(“*pesquisa metodológica em enfermagem*” OR “nursing methodology research”) AND (“*enfermagem*” OR “nursing”) AND (“*tecnologia educacional*” OR “educational technology” OR “*tecnologia*” OR “technology” OR “*tecnologias*” OR “technologies”) AND (db:(“LILACS” OR “BDENF”)) AND (year_cluster: [2011 TO 2023])
CINAHL	(“nursing methodology research”) AND (“nursing”) AND (“educational technology” OR “technology” OR “technologies”)
EMBASE	(‘nursing methodology research’/exp OR ‘nursing methodology research’) AND (‘nursing’/exp OR ‘nursing’) AND (‘educational technology’/exp OR ‘educational technology’ OR ‘technology’/exp OR ‘technology’ OR ‘technologies’) AND [2011-2023]/py AND ([english]/lim OR [portuguese]/lim OR [spanish]/lim)
MEDLINE/ PubMed	(“nursing methodology research”[All Fields]) AND (“nursing”[All Fields]) AND (“educational technology”[All Fields] OR “technology”[All Fields] OR “technologies”[All Fields])) AND (2011:2023[pdat])
Scopus	TITLE-ABS-KEY (“nursing methodology research”) AND (“nursing”) AND (“educational technology” OR “technology” OR “technologies”)) AND PUBYEAR > 2010 AND PUBYEAR < 2024
Web of Science	“nursing methodology research” (All Fields) AND “nursing” (All Fields) AND “educational technology” OR “technology” OR “technologies” (All Fields)

In the fourth stage, definitive search and data collection, the results were exported in the Research Information Systems (RIS) format to the Rayyan^®^ review manager software^(14)^. In the fifth stage, duplicate documents were detected and excluded, and the remaining articles were analyzed by reading titles and abstracts by three researchers, one specialist and two PhD holders, independently, responsible for selection in two stages, with masking, from the perspective of maintaining methodological rigor and double-blind review.

Conflicts between these were decided by a third researcher with a PhD, called upon to define selection. JabRef^®^ and Microsoft Excel^®^ were used to build the bibliographic portfolio. In the sixth stage, Impact Factor, year of publication and number of citations via Google Scholar^®^ were identified. In the seventh stage, the *InOrdinatio* value of each article was generated using the Rankln version 2.0 spreadsheet. This value was acquired using the *InOrdinatio* 2.0 formula = {(∆* IF) - [λ* (ResearchYear - PubYear)/HalfLlife] + Ω* ∑ Ci/[(ResearchYear+1) -PubYear]}^(15)^. Thus, 10 (greatest importance) was established for the constants ∆ (Impact Factor), λ (year of publication), Ω (number of citations). In this way, the articles were classified in descending order according to the result obtained.

The eighth stage was carried out simultaneously with the sixth stage, in which the selected articles were downloaded. The ninth stage was partially met, as only a skimming reading of the articles selected to compose the bibliographic portfolio of this review was carried out, as reading the studies in full is not the objective of bibliometric research.

### Analysis of results, and statistics

To analyze the results, the three classic laws of bibliometrics were applied: I) Lotka’s Law: to analyze author productivity; II) Bradford’s Law: to assess journal productivity; III) Zipf’s Law: to assess keyword frequency^(8)^.

The data was organized in Microsoft Excel^®^ spreadsheets. The data were analyzed descriptively and, to represent the findings of Lokta’s and Zipf’s Laws, the Visualizing Scientific Landscapes (VOSviewer^®^) version 1.6.19 was used, which consists of a representation referring to the occurrence of terms as well as their relationships, in which the thickness of the connections indicates the intensity of cooperation between the terms^(16)^.

## RESULTS

The initial search, according to the inclusion criteria, resulted in a total of 346 studies, which came from the following databases: BDENF (25); CINAHL (7); EMBASE (1); LILACS (25); PubMed/MEDLINE (159); Scopus (124); and Web of Science (5). In Rayyan^®^, 124 duplicate articles were identified and excluded, leaving 222 studies as well as information on the main publication journals, authors and language of origin; these were part of the scope to be analyzed according to Lotka’s, Bradford’s and Zipf’s Laws. After reading the title and abstract of the 222 articles in Rayyan^®^, 196 were excluded because they were not closely linked to the topic. Thus, the final selection of studies to compose the bibliographic portfolio was 26 articles to be analyzed by *Methodi Ordinatio*.

It is noteworthy that the studies were distributed in 76 journals, with emphasis on Nurse Educator (42; 19%), Computers, Informatics, Nursing (15; 6.8%), Nurse Education in Practice (10; 4.51%), Journal of Nursing Education (9; 4%), Nurse Education Today (9; 4%), *Revista Brasileira de Enfermagem* (8; 3.6%), Midwifery (6; 2.7%), Journal of Advanced Nursing (5; 2.25%), Nurse Researcher (5; 2.25%) and Practising Midwife (5; 2.25%). Together they total 114 studies, corresponding to approximately 51.23% of studies. The details of this distribution can be found in Table 1, in which the journals described represent quadrants 1 and 2.

**Table 1:** Scientific production on nursing technology construction, validity and application according to Bradford’s, Lokta’s and Zipf’s Laws. 2011-2023

Bradford’s Law			
**Quadrant**	**Journals**	**Studies**	**%**
1	2	57	25.68
2	8	57	25.68
3	20	54	24.32
4	46	54	24.32
Total	76	222	100
**Lokta’s Law**			
**Number of authors**	**% authors**	**Number of studies**	**% studies**
130	20	178	25.39
571	80	523	74.61
Total	100	701	100
**Zipf’s law**			
**Zone**	**Total words**	**Frequency**	**%**
Trivial	22	1482	41.89
Interest	192	1225	34.63
Noise	697	830	23.48
Total	911	3537	100

The total number of authors was 653, responsible for 701 authors, and the number of documents according to the number of authors was as follows: one author (Charalambous, A) published four articles; two authors published three articles; 41 authors published two articles; and 609 authors published only one article. Hence, 20% of authors accounted for 25.39% of studies, as explained in Table 1.

In relation to the language of studies, there was a predominance of studies in English (208; 93.69%), followed by Portuguese (12; 5.41%), German (1; 0.45%) and Spanish (1; 0.45%). Regarding the number of articles published per year, we have the following numbers: 2011 (27 - 12.16%); 2012 (43 - 19.37%); 2013 (31 - 13.96%); 2014 (26 - 11.71%); 2015 (16 - 7.21%); 2016 (15 - 6.76%); 2017 (14 - 6.31%); 2018 (14 - 6.31%); 2019 (16 - 7.21%); 2020 (9 - 4.05%); 2021 (5 - 2.25%); 2022 (4 - 1.80%); and 2023 (2 - 0.90%). The year 2012 stood out with the highest number.

**Figure 1:**
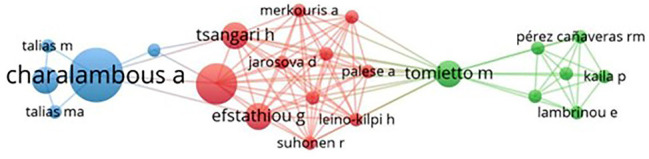
Co-authorship network for scientific production on nursing technology construction, validity and application. 2011-2023

Figure 1 shows the analysis of co-authorship network. Using the filter of at least two occurrences of authors in articles, 14 clusters and a total of 642 items were generated, with 23 connections between the authors. The scientific influence of certain authors is observed, responsible for concentrating the focus of scientific productions and for greater numbers of connections with others.

Regarding keywords, 911 terms were found, with a general frequency of 3,537 repetitions, being categorized into three zones, such as trivial information (22), interesting information (192) and noise (697) according to the number of repetitions. Table 1 shows the details of the result of Zipf’s Law.

**Figure 2:**
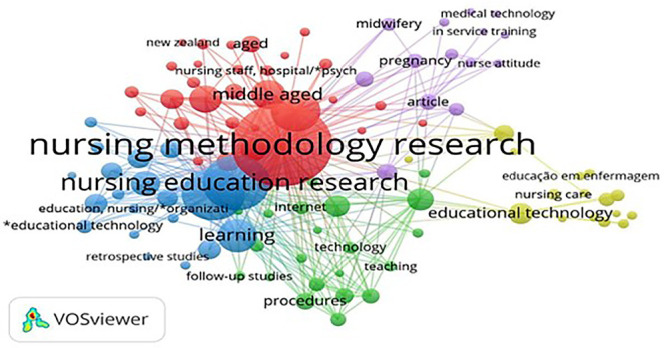
Distribution network of keywords in scientific production on nursing technology construction, validity and application. 2011-2023

The analysis of the distribution of keywords was carried out using VOSviewer^®^ and is represented in Figure 2. In this assessment, the filter of at least two word occurrences in articles was used, causing the appearance of 351 clusters, of which were 26 were selected to compose this review. In Figure 2, the arrangement of terms in Portuguese and English can be seen, of which the keyword nursing methodology research stood out, with 179 occurrences in the texts.

Chart 2 characterizes the profile of the 26 selected studies classified according to *InOrdinatio*, based on the journal’s Impact Factor (IF), number of citations (CI) and year of publication. Only three studies (1, 2 and 3) have *InOrdinatio* greater than 80, i.e., a better bibliographic portfolio of the state of the art, and, of the 26 studies, only two have *InOrdinatio* less than one.

**Chart 2:** Characterization of scientific production on nursing technology construction, validity and application according to *InOrdinatio*. 2011-2023

ID	Title	Authors	Journal	Year of publication	CI	IF	*InOrdinatio*
1^(17)^	*Evaluation of vSim for Nursing in* an *Adult Health Nursing Course: A Multisite Pilot Study.*	Wright RR *et al*.	CIN: Computers, Informatics, Nursing	2018	43	2.1	86.08772
2^(18)^	Validation of an educative manual for patients with head and neck cancer submitted to radiation therapy	Cruz FOAM *et al*.	*Revista Latino-Americana de Enfermagem*	2016	75	-	84.53947
3 ^(19)^	Validation of educational material for the care of people with intestinal stoma	Sena JF *et al*.	*Revista Latino-Americana de Enfermagem*	2020	34	-	81.05263
4 ^(20)^	Mobile health technology for gestational care: evaluation of the GestAção’s app	Silva RM *et al*.	*Revista Brasileira de Enfermagem*	2019	30	1.3	67.73684
5 ^(21)^	*Jogo educativo de administração de medicamentos: um estudo de validação*	Moreira APA *et al*.	*Revista Brasileira de Enfermagem*	2014	54	1.3	55.15789
6 ^(22)^	*Construção de hipermídia para apoio ao ensino da sistematização da assistência de enfermagem*	Salvador PTCO *et al*.	*Revista Gaúcha de Enfermagem*	2019	23	1.1	51.73684
7 ^(23)^	Educational hypermedia in nursing assistance at birth: building and validation of content and appearance	Oliveira LL *et al*.	*Revista Brasileira de Enfermagem*	2019	21	1.3	49.73684
8 ^(24)^	Assessment of a prototype for the Systemization of Nursing Care on a mobile device	Rezende LCM *et al*.	*Revista Latino-Americana de Enfermagem*	2016	39	-	39.53947
9 ^(25)^	Structuring methodology of the Computerized Nursing Process in Emergency Care Units	Paese F *et al*.	*Revista Brasileira de Enfermagem*	2018	18	1.3	36.42105
10 ^(26)^	*Desenvolvimento de Ambiente Virtual de Aprendizagem em Enfermagem sobre ressuscitação cardiorrespiratória em neonatologia*	Rodrigues RCV *et al*.	*Revista da Escola de Enfermagem da USP*	2013	42	1.1	36.02392
11 ^(27)^	Validation of an educational manual for breast cancer patients undergoing radiotherapy	Cruz FOAM *et al*.	*Revista Latino-Americana de Enfermagem*	2020	15	-	33.55263
12 ^(28)^	Plataforma PEnsinar^®^: a learning tool for teaching the nursing process	Melo ECA *et al*.	*Revista Brasileira de Enfermagem*	2018	16	1.3	33.08772
13 ^(29)^	*Construção e validação de uma tecnologia educacional para prevenção da sífilis congênita*	Costa CC *et al*.	*Acta Paulista de Enfermagem*	2020	10	1.1	32.05263
14 ^(30)^	Software for systematization of nursing care in medical units	Silva Junior MG *et al*.	*Revista Brasileira de Enfermagem*	2018	14	1.3	29.75439
15 ^(31)^	*Construção e validação de videoaula sobre ressuscitação cardiopulmonar*	Alves MG *et al*.	*Revista Gaúcha de Enfermagem*	2019	12	1.1	29.73684
16 ^(32)^	T-NDX Diagram: Educational Technology Used to Teach Diagnostic Reasoning Based on Nursing Theories.	Lopes ROPR *et al*.	International Journal of Nursing Knowledge	2020	4	2.1	27.05263
17 ^(33)^	Soulage-tavie: Development and validation of a virtual nursing intervention to promote self-management of postoperative pain after cardiac Surgery	Martorella G, Côté J, Choinière M	CIN: Computers, Informatics, Nursing	2013	16	2.1	22.38756
18 ^(34)^	Validation of a booklet for the correct use of personal protective equipment in the context of COVID-19	Silva ABP *et al*.	*Texto & Contexto Enfermagem*	2021	3	1.4	21.36842
19 ^(35)^	Virtual Guide On Ocular Self-Examination To Support The Self-Care Practice For People With HIV/AIDS	Lima MA *et al*.	*Revista da Escola de Enfermagem da USP*	2014	19	1.1	18.15789
20 ^(36)^	Development of a mobile application focusing on developmental support care for Korean infants born prematurely: a methodological study.	Park JH, Cho H	Child Health Nursing Research	2022	1	0.9	12.68421
21 ^(37)^	Construction of a nursing care protocol for children in post-hematopoietic stem cell transplantation	Rodrigues J *et al*.	*Revista Gaúcha de Enfermagem*	2022	0	1.1	9.684211
22 ^(38)^	A clinical nurse specialist intervention to facilitate safe transfer from ICU	St-Louis L, Brault D	Clinical Nurse Specialist (CNS)	2011	33	-	9.595142
23 ^(3)^	*Tecnologia educacional para pessoas com doença renal crônica: construção e validação de conteúdo*	Santos FGT *et al*.	*Revista de Pesquisa*	2021	3	-	7.368421
24 ^(39)^	*Análise de qualidade de objeto virtual de aprendizagem para avaliação da dor em enfermagem*	Alvarez AG *et al*.	*Revista Cubana de Enfermería*	2018	4	0.3	3.087719
25 ^(40)^	*Construção e avaliação de um ambiente virtual de aprendizagem para liga de segurança do paciente*	Moraes AIS *et al*.	*CuidArte Enfermagem*	2021	1	-	0.701754
26 ^(41)^	*Avaliação de objeto virtual de aprendizagem sobre raciocínio diagnóstico: um estudo descritivo*	Costa CPV *et al*.	Online Brazilian Journal of Nursing	2015	5	0.1	-3.97076

*Caption: ID - identification; IF - Journal Impact Factor; CI - number of citations.*

The main study topic was the Nursing Process (NP) (6; 23%), followed by nursing fundamentals (12%) and infectious diseases (12%), with three studies each. The topics surgical nursing (8%), neonatology (8%), obstetrics (8%) and oncology (8%) had two studies each. The topics with one publication were hematology (4%), nephrology (4%), adult health (4%), patient safety (4%), nursing theories (4%) and emergency (4%).

The following technological artifacts were described in the studies: software (7; 27%), virtual environment (6; 23%), booklet (5; 19%), hypermedia (2; 8%), manual (2; 8%), instrument (1; 4%), game (1; 4%), protocol (1; 4%) and video lesson (1; 4%). Regarding the steps described, 13 studies (50%) presented technology construction and validity, eight (31%) detailed construction, three (12%) presented application and two (8%) presented validity.

## DISCUSSION

Less than ten years ago, innovation and technological development were still an incipient scientific practice in nursing. However, it is known that the proposition and use of technologies enable improvements in quality of health care, with better cost-effectiveness and efficiency, as knowledge networks are used to build products arising from their practice and that provide technological innovation to be closer to users’ reality^(5)^.

On the other hand, methodological studies debuted in nursing in 2006 and showed a considerable increase in studies from 2015^(42)^, making it imperative to develop technologies in their different stages, such as creation, validity and application of products and processes for different purposes^(6)^.

It is noteworthy that the largest percentage of productions present in this study corresponds to research published in English and with exponential growth over the last two decades, with the majority of studies published in 2012. Technological development began to have a strong impact on production of scientific knowledge, in nursing and health, taking into account the multiple individual and collective demands that arise in health care and education^(8)^.

Furthermore, English is also a universal language for science, as it allows all researchers around the world to communicate and exchange information using a single language. As a consequence of this act, there has been an increase in studies in English over the years as a reflection of efforts of scientists, educational institutions and journals with a view to internationalizing scientific studies^(43)^.

As a result of studies assessed by the *InOrdinatio* method, the majority of studies are from Brazilian authors. However, they also have translation and dissemination of their texts into English, which reinforces the strategy of using English as a measure of internationalization of Brazilian scientific productions, in addition to increasing Impact Factor, greater possibility of reading and citation when greater access to studies is provided^(44)^.

Expanding access to studies is directly related to greater qualification and prestige of journals so that nursing production advances in quality and visibility. Prestigious journals seek established vehicles, such as important databases, libraries and scientific repositories, to disseminate their scientific productions with a view to ensuring recognition and dialogue between the scientific community in different fields of interest^(45)^.

Furthermore, the results of this review point to a concentration of authorship, given that one author published four articles and 609 authors published only one article each. This finding supports Lotka’s Law, which describes the pattern of authors’ scientific production, i.e., it states that few authors, authorities on a subject, produce a lot, and many authors, probably with less prestige and a broad spectrum of study, produce little on the same topic. This law allows checking the productivity of authors and the most developed research centers in a given area^(46-47)^.

Studies that identify productivity and centers of excellence in a given area are important for consolidating and strengthening this field of investigation. This perspective is highlighted in a documentary study that draws attention to the adoption and conduct of lines of research in nursing that involve and present similar themes in scientific research with investigative traditions, produce projects with affinities between them and with a greater direction of production, contributing to systematization of scientific knowledge^(48)^.

Concerning Zipf’s Law, it was shown that a smaller number of words were cited many times and that many words were cited few times, therefore, an inversely proportional result. As expected, the word “nursing methodology research” had the highest occurrence, followed by elements that are components, such as “nursing research education” and “learning”, since the study aims to analyze the bibliometric profile of scientific production on nursing technology construction, validity and application.

Methodological studies enable investigation to obtain and organize data to construct, validate and assess, according to rigorous methodological precepts, such as research methods, to develop reliable instruments that have applicability in different realities and in different areas of knowledge, such as nursing, with a view to creating and validating different technological products, such as educational technologies (ET)^(49)^.

The type of methodological study was the most discussed in the research found, coming mainly from construction and validity studies. The first phase of methodological studies (production) is built based on scientific evidence present in the literature, proceeding with the review of studies available in the literature and evidence of reality, which requires primary studies both for situational diagnosis and to promote shared construction and facilitate constructing technology^(50)^.

In relation to the areas of knowledge, it was identified that technology application was more related to NP. Technology use promotes adherence to NP by nurses when compared to rates before the use of technologies^(51)^. Furthermore, nursing’s interest in implementing systematized and quality care is evident as well as the rigor in complying with Resolution 358 of 2009 of the Federal Nursing Council (COFEN - *Conselho Federal de Enfermagem*) in making the use of NP mandatory in public or private health services^(52)^.

Technologies, when developed for educational use, are endowed with knowledge that aims to produce/prepare, apply/intervene and monitor an educational process. Furthermore, they are instruments that enable assessing health education, as they enable health actions, supported by ET, that come closer to individuals’ reality and promote a higher quality of life^(51)^. The act of creating an ET based on scientific evidence allows the target audience to have access to a cluster of content made available in an easy, practical and current way, with the provision of safe information in relation to the content covered^(53)^.

It is assumed that technology production arises from the perception of the need understood in discussion, sharing of knowledge and creativity encouragement^(54)^. This reflects in the increase in production related to ET construction and validity, as they constitute benefits for the population with a significant improvement in quality of life, promoting the adoption of healthy behaviors, transformation of practices, innovation in the way of learning to learn and strengthening meaningful learning with autonomy and empowerment^(55-56)^.

The validity stage is related to different aspects, such as validating content, appearance, semantics, usability, applicability, playfulness, interactivity, among other actions. It is considered that there is the possibility of different interactions at the time of validity, which requires planning to develop the steps, which can be concomitant or sequential^(6)^. Making a technology with an educational focus valid means formalizing the relevance of content developed from the perspective of encompassing information that is suitable and safe for use in an educational manner, following the required methodological precepts^(57)^.

Therefore, it is important that, after creating an ET, the next step is validity; this step is carried out by specialists with expertise in the area of study presented. The Ministry of Health (MoH) aims that technology production can contain clear, accessible, intelligible language and appropriate to the target audience’s reality, as it can help or hinder the transmission of the desired message; therefore, the experience of experts is essential for ET to be considered valid^(58)^.

With regard to the technological artifact produced, there was a prevalence of software, which is a logical set of information that uses algorithms for data processing and which results in a type of program^(59)^. In the educational context, these artifacts promote new learning experiences and, when used to compose educational material, they demonstrate an innovative practice in the teaching and learning process^(60)^.

### Study limitations

Assembling search string represented a critical step, since the inappropriate choice of descriptors could result in exclusion of relevant information. Furthermore, the number of studies associated with the search strings may be a limitation, as a small number may indicate a very restrictive search. These limitations made the development and approach careful and judicious in conducting the research, aiming to mitigate these obstacles and ensure the integrity and relevance of results.

### Contributions to nursing, health or public policy

The present study explores what has been produced by nursing regarding the development of technologies, especially those based on consolidated scientific methods and well described in the literature. Therefore, this research carries data that can support new methodological studies, in addition to bringing insights that enable creating collaborative networks for technology production.

## CONCLUSIONS

Technology development plays a fundamental role in consolidating nursing as a science. It is through the process of construction and validity of what is created for the profession that the class autonomy becomes evident. This study evokes a variety of strategies adopted by the authors of the works analyzed in the search for developing their technologies. This strengthens the characteristic of plurality present in the theme of technological productions: the infinity of possibilities to be developed.

There is still a strong appeal for ET production. The tendency to develop products aimed at teaching nursing, especially related to information technologies (software, applications and IT support), is a worldwide characteristic and is present in the different contexts in which the profession operates, such as clinical practice, patient management, care and improvement of general institutional processes.

With the data from this profile, the potential for future studies also stands out, since the appreciation of the topic of bibliometric production leads to insights capable of elucidating how technological production in nursing occurs. It is recommended that further research be carried out, considering further specifying the branches through which nursing technology production can permeate, exploring its types-classifications and other aspects.

## Supplementary Material

0034-7167-reben-77-03-e20230452-suppl01

## Data Availability

The protocol for this study was registered on the Figshare platform (https://figshare.com/s/4cc25bdcc1711a586f64).
