# Supplementary material for: Profile of scientific production on nursing technology construction, validity and application: a bibliometric study
Source: Rev Bras Enferm. 2024 Jul 29;77(3):e20230452. doi: 10.1590/0034-7167-2023-0452 (PMC11290734; doi:10.1590/0034-7167-2023-0452)
Supplement: 0034-7167-reben-77-03-e20230452-suppl01 [file 0034-7167-reben-77-03-e20230452-suppl01.pdf]

PROTOCOLO DE REVISÃO BIBLIOMÉTRICA

Autores: Fernando Conceição de Lima; Taís dos Passos Sagica; João Lucas Moraes de Souza; Mary Elizabeth de Santana; Marta Lenise do Prado; Ivonete Vieira Pereira Peixoto; Rubenilson Caldas Valois.

1. Enfermeiro. Mestrando em enfermagem pelo Programa de Pós-graduação em Enfermagem com área de concentração em Enfermagem no contexto da sociedade amazônica com linha de pesquisa educação e cuidado em saúde e enfermagem na Amazonia PPGENF-UEPA/UFAM. ORCID: https://orcid.org/0000-0002-9418-3711.
2. Enfermeira. Mestrando em enfermagem pelo Programa de Pós-graduação em Enfermagem com área de concentração em Enfermagem no contexto da sociedade amazônica com linha de pesquisa educação e cuidado em saúde e enfermagem na Amazonia PPGENF-UEPA/UFAM. ORCID: https://orcid.org/0000-0002-6871-0100.
3. Enfermeiro. Mestrando em enfermagem pelo Programa de Pós-graduação em Enfermagem com área de concentração em Enfermagem no contexto da sociedade amazônica com linha de pesquisa educação e cuidado em saúde e enfermagem na Amazonia PPGENF-UEPA/UFAM. ORCID: https://orcid.org/0000-0003-3652-751X.
4. Enfermeira. Doutora. Professora Titular do Curso de Enfermagem da Universidade do Estado do Pará (UEPA) e Professora Associada I da Faculdade de Enfermagem da Universidade Federal do Pará (UFPA). ORCID: https://orcid.org/0000-0002-3629-8932.
5. Enfermeira. Doutora. Professora Visitante na Universidade do Estado do Pará - UEPA - Bolsista FAPESPA (2021-2023). Docente permanente no Programa de

Pós-Graduação em Enfermagem - modalidade acadêmico associado/UFAM-UEPA. Professora titular aposentada da Universidade Federal de Santa Catarina. Professora Visitante na Escola de Enfermagem de Manaus/UFAM (2019-2021). Docente voluntária do Programa de Pós-graduação em Enfermagem (Mestrado e Doutorado) da UFSC (2015-2020). Bolsista CNPq 2005-2010 (PQ 2); 2011-2012 (PQ 1D) e 2014-2019 (Pq 1B). ORCID: https://orcid.org/0000-0003-3421-3912.

6. Enfermeira. Doutora. professora Adjunto da Universidade do Estado do Pará, Diretora do Centro de Saúde Escola do Marco CCBS/UEPA e Vice-Coordenadora do Mestrado e Doutora do programa ESA/UEPA. ORCID: https://orcid.org/0000-0002-5463-9630.
7. Enfermeiro. Doutor. Professor Adjunto I da Universidade do Estado do Pará (UEPA); Professor permanente programa de pós graduação em Enfermagem associado UEPA/UFAM. ORCID: https://orcid.org/0000-0001-9120-7741.

TITULO: perfil bibliométrico da produção científica sobre construção, validação e aplicação de tecnologias na enfermagem.

INTRODUÇÃO E JUSTIFICATIVA

O processo de construção de aparatos tecnológicos permeia diversos formatos e moldes. A pesquisa metodológica é o tipo que se volta para o desenvolvimento de produtos e costuma envolver métodos complexos. É uma metodologia que pode ainda ser aplicada às investigações dos formatos de obtenção e organização de dados, além da condução de pesquisas rigorosas. [1,2]

Ela é capaz de lidar com o desenvolvimento, a validação e a avaliação de ferramentas e métodos de pesquisa. Neste tipo de estudo, o pesquisador tem como meta a elaboração de um produto confiável e utilizável que possa ser empregado por outros pesquisadores e outras pessoas. Este método é aplicável em qualquer área científica, lidando com fenômenos complexos, como o comportamento ou a saúde dos indivíduos. [1,2]

No campo da Enfermagem, há uma grande quantidade de estudos metodológicos, com vistas à produção de tecnologias assistenciais, gerenciais e/ou educacionais. Além destas, é comum encontrar propostas de avaliação e adaptação cultural de instrumentos internacionais para a utilização no país de interesse. [3,4] De modo geral, o enfoque dos estudos metodológicos se volta para o desenvolvimento de constructos aplicáveis na ciência e na profissão de enfermagem. [5]

Como característica principal, estes estudos apresentam-se em forma de processos encadeados, iterativos e interativos, nos quais repousam os estágios de desenvolvimento de uma tecnologia. Não há consenso entre autores sobre as melhores nomenclaturas das fases, ou até mesmo sobre o quantitativo destas, porém, assumem-se atualmente alguns modelos de divisão, como a realização de diagnóstico situacional, as organizações de revisões de literatura, a construção da tecnologia, a validação e a testagem. [6,3]

De modo amplo, o termo "tecnologia" pode se referir a técnicas, métodos, instrumentos, procedimentos e equipamentos que promovem a execução e geram produtos e serviços. Na área da saúde, sobretudo no que se refere à prestação de serviços de enfermagem, a adoção de tecnologias é capaz de refinar as práticas profissionais, as relações interpessoais e o gerenciamento dos processos dentro dos serviços. [2,6] Desse modo, a aplicação de tecnologias ocorre de diversas maneiras e tem o seu significado atribuído de acordo com a sua utilização. [3]

OBJETIVO

Analisar o perfil bibliométrico da produção científica sobre construção, validação e aplicação de tecnologias na enfermagem.

MÉTODOS

ASPECTOS ÉTICOS

A pesquisa não foi submetida à apreciação em Comitê de Ética em Pesquisa com Seres Humanos, haja vista que foi realizada com dados secundários e de domínio público. No entanto, preservou-se os direitos autorais dos estudos.

DESENHO, PERÍODO E LOCAL DO ESTUDO

Trata-se de um estudo bibliométrico acerca da construção, validação e aplicação de tecnologias na enfermagem. Este tipo de método caracteriza-se por evidenciar métricas, indicadores, produção e a disseminação científica de determinado tema. [7]

Fundamentou-se na *methodi ordinatio*, uma ferramenta multicritério de auxílio que permite ordenar os artigos considerando três fatores: número de citações, fator de impacto (métrica) e ano de publicação. [8] Além das diretrizes do fluxograma do *Preferred Reporting Items for Systematic Reviews and Meta-Analyses* (PRISMA) [9], conforme figura 1.

Figura 1 – Fluxograma de seleção dos estudos na RI, Belém, Pará, Brasil, 2023.

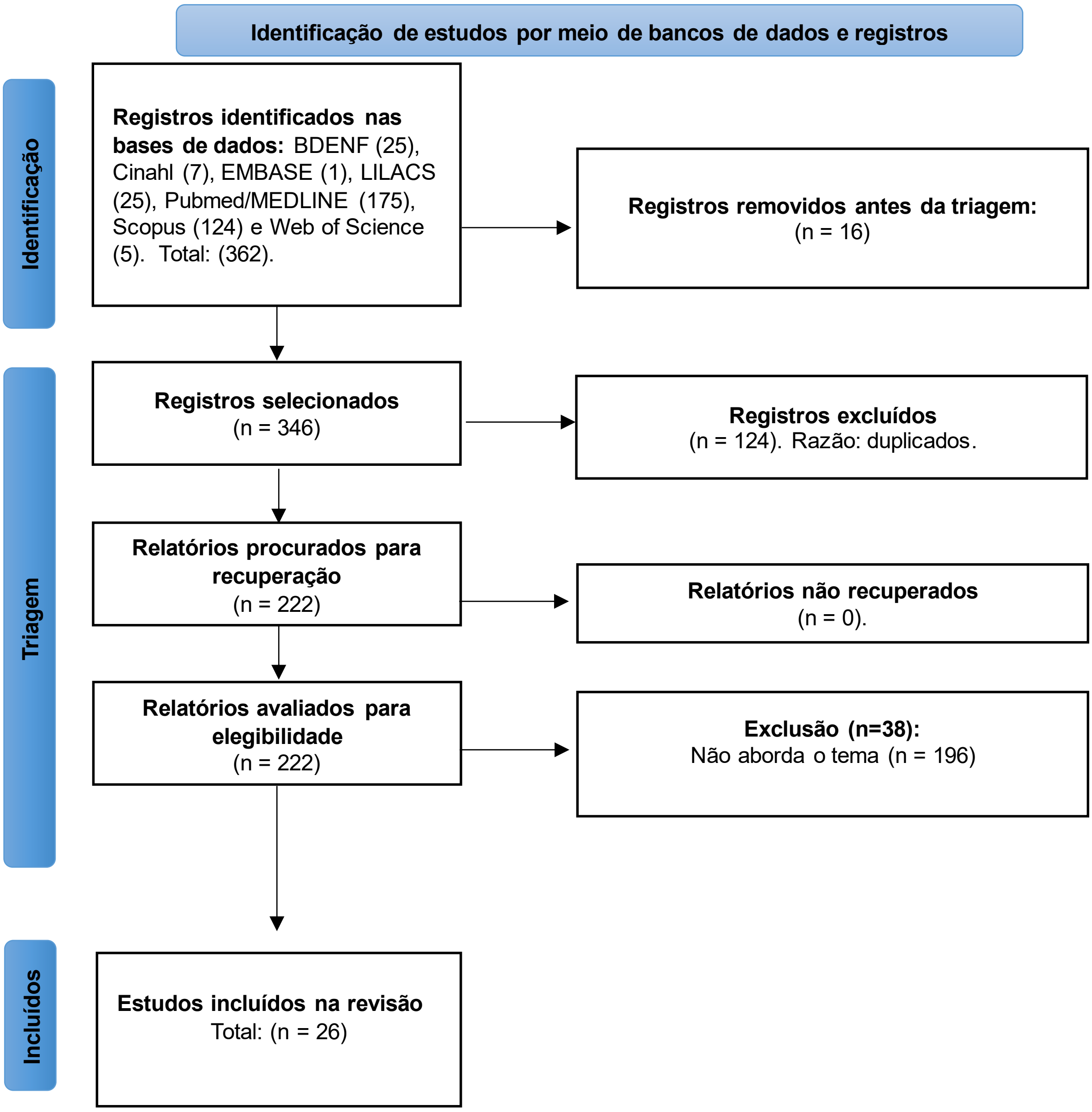

Fonte: adaptado (Page MJ et al, 2020).

Este estudo Desta forma, este estudo seguiu as nove etapas propostas: 1) definição da intenção da pesquisa; 2) pesquisa preliminar nas bases de dados; 3) definição e combinação das palavras-chave e bases de dados; 4) busca definitiva e coleta de dados; 5) procedimento de filtragem; 6) identificação do fator de impacto, ano de publicação e número de citações; 7) classificação dos artigos no *InOrdinatio* utilizando a planilha *Rankln*; 8) Localização dos textos em formato integral; 9) Leitura sistemática e análise dos artigos. [8]

Foi realizada a busca preliminar nas seguintes bases de dados: Base de Dados de Enfermagem (BDENF), *Cummulative Index to Nursing and Allied Health* (CINAHAL), EMBASE; Literatura Latino-Americana e do Caribe em Ciências da Saúde (LILACS), MEDLINE, via *National Library of Medicine* (PubMed), Scopus e *Web of Science* (WoS). Os dados foram coletados no mês de maio a abril a junho de 2023.

POPULAÇÃO OU AMOSTRA; CRITÉRIOS DE INCLUSÃO OU EXCLUSÃO

Na primeira etapa, utilizou-se a estratégia PICo [11] para formular a seguinte questão de pesquisa: "qual o perfil das produções científicas sobre construção, validação e aplicação de tecnologias em Enfermagem?", sendo P (população): construção, validação e aplicação de tecnologias; I (interesse): perfil das produções científicas; e Co (contexto): Enfermagem.

Foram estabelecidos os seguintes critérios de inclusão: artigos científicos sobre a construção, validação e/ou aplicação de tecnologias na enfermagem, disponibilizados em português, espanhol e inglês. Utilizou-se o recorte temporal a partir de 2011, pois neste ano houve o crescimento de publicações (teses e dissertações) acerca do tema na área. [12] Foram excluídos: artigos duplicados e que não respondam à questão de pesquisa.

PROTOCOLO DO ESTUDO

Após a segunda etapa de pesquisa preliminar nas bases de dados, deu-se início a busca dos estudos por meio do acesso remoto ao conteúdo da Comunidade Acadêmica Federada (CAFe), um recurso disponibilizado pela Coordenação de Aperfeiçoamento de Pessoal de Nível Superior (CAPES) e Ministério da Educação (ME) e assinado pela Universidade do Estado do Pará (UEPA), por meio da combinação dos Descritores em Ciências da Saúde (DeCS)/ Medical Subject Headings (MeSH)/Emtree (vinculado ao Embase Index), com o auxílio dos operadores booleanos AND e OR.

Na terceira etapa, para a definição das palavras-chave, utilizaram-se os idiomas português e inglês com a seguinte combinação: ("pesquisa metodológica em enfermagem" OR "nursing methodology research") AND ("enfermagem" OR "nursing") AND ("tecnologia educacional" OR "educational technology" OR "tecnologia" OR "technology" OR "tecnologias" OR "technologies"). Esta estratégia de busca foi padronizada nas bases de dados e portais eletrônicos: Base de dados de Enfermagem (BDENF), Literatura Latino-americana e do Caribe em Ciências da Saúde (LILACS), Medical Literature Analysis and Retrieval System Online (MEDLINE) via PubMed Central® (PMC), Scopus Info Site (Scopus), Web of Science (WOS), EMBASE e CINAHL Ultimate, em que se utilizou a ferramenta de pesquisa avançada e o rótulo de campo da pesquisa baseado em tópicos (título, resumo e palavras-chave).

Na quarta etapa, busca definitiva e coleta de dados, os resultados foram exportados no formato *Research Information Systems* (RIS) para o *software* gerenciador de revisões Rayyan®. [(13)] Na quinta etapa, foram detectados e excluídos os documentos duplicados e os demais artigos foram analisados por meio da leitura dos títulos e resumos, por três pesquisadores, sendo um especialista e dois doutores de maneira independente, responsáveis pela seleção em duas etapas, com mascaramento, na perspectiva de manter o rigor metodológico e a revisão dupla-cega.

Os conflitos entre estes foram decididos por um terceiro pesquisador, com título de doutor, acionado para definir a seleção. Foram utilizados os programas JabRef® e o *software Microsoft Excel®* para a construção do portfólio bibliográfico. Na sexta etapa, houve a identificação fator de impacto, ano de publicação e número de citações pelo *Google Scholar*. Na sétima etapa, houve a geração do valor InOrdinatio de cada artigo utilizando a planilha Rankln versão 2.0. Este valor foi adquirido por meio da fórmula $InOrdinatio2.0 = \{(\Delta^* \ IF) - [\lambda^* \ (ResearchYear - PubYear)/HalfLlife] + \Omega^* \sum Ci/[(ResearchYear+1) - PubYear]\}$. [(14)] Foi estabelecido 10 (maior importância) para as constantes Δ (fator de impacto), λ (ano de publicação), Ω (número de citações). Dessa forma, os artigos foram classificados de forma decrescente conforme o resultado obtido.

A oitava etapa, foi realizada concomitantemente à sexta etapa, na qual foi realizado o *download* dos artigos selecionados. A nona etapa foi atendida parcialmente, por ter sido realizada apenas a leitura flutuante dos artigos selecionados para compor o portifólio bibliográfico desta revisão, visto que a leitura na íntegra dos estudos não é o objetivo da pesquisa bibliométrica.

ANÁLISE DE DADOS

Para a análise dos resultados serão aplicadas as três leis clássicas da bibliometria: I) Lei de Lotka: para analisar a produtividade dos autores; II) Lei de Bradford: visando avaliar a produtividade dos periódicos; III) Lei de Zipf: para avaliar a frequência de palavras-chave. (12)

A organização dos dados se dará em planilhas do *Microsoft Excel*®. Os dados serão analisados descritivamente e para a representação dos achados da lei de Lokta e Zipf, utilizou-se o *software Visualizing scientific landscapes* VOSViewer® versão 1.6.19 que consiste em uma representação referente a ocorrência dos termos, bem como suas relações, na qual a espessura das conexões indica a intensidade da cooperação entre os termos. (15)

REFERÊNCIAS

1. Polit DF, Beck CT. The content validity index: Are you sure you know what’s being reported? Critique and recommendations. Res Nurs Health. 2006;29(5):489–97. https://doi.org/10.1002/nur.20147

2.Lacerda MR, Ribeiro RP, Costenaro RGS. Metodologias da pesquisa para a Enfermagem e Saúde: da teoria à prática volume II. Porto Alegre: Moriá; 2018. 151-76 p.

3. Santos FGTD, Laqui VDS, Sanches RDCN, Rêgo ADS, Salci MA, Radovanovic CAT. Educational technology for people with chronic renal disease: construction and validation of content. R.pesq:cuid.fundamOnline [Internet]. 2021 [cited 2023 Oct 10];13: 517–23. Available from: http://seer.unirio.br/cuidadofundamental/article/view/9263

4.Teixeira E. Participatory interfaces in methodological research for nursing investigations. Rev. enferm. UFSM. 2019;9:e1. https://doi.org/10.5902/2179769236334

5. Ferreira DS, Ramos FRS, Teixeira E. Mobile application for the educational praxis of nurses in the Family Health Strategy: ideation and prototyping. Esc. Anna Nery. 2021;25(1):e20190329. https://doi.org/10.1590/2177-9465-EAN-2019-0329

6.Teixeira E, Nascimento MHM. Projetos de continuidade: uma possibilidade para a implementação de soluções tecnológicas. Online braz j nurs [Internet]. 10 de fevereiro de

2023 [citado 28 de setembro de 2023];22. Disponível em: https://www.objnursing.uff.br/index.php/nursing/article/view/6604.

7.Soares SV, Picolli IRA, Casagrande JL. Bibliographic Research, Bibliometric Research, Review Article and Theoretical Test in Administration and Accounting. RAEP. 2018;19(2):308–39. https://doi.org/10.13058/raep.2018.v19n2.970

8. Pagani RN, Kovaleski JL, Resende LM. Methodi Ordinatio: a proposed methodology to select and rank relevant scientific papers encompassing the impact factor, number of citation, and year of publication. Scientometrics. 2015;105(3):2109–35. https://doi.org/10.1007/s11192-015-1744-x

9. Page MJ, McKenzie JE, Bossuyt PM, Boutron I, Hoffmann TC, Mulrow CD, et al. The PRISMA 2020 statement: an updated guideline for reporting systematic reviews. BMJ. 2021;372:71. https://doi.org/10.1016/j.ijsu.2021.105906

10. Lima FC de, Prado ML do, Sagíca T dos P, Lucas Moraes de Souza J, Santana ME de, Caldas Valois R. Protocolo: Perfil da produção científica sobre construção, validação e aplicação de tecnologias na enfermagem. [Internet]. figshare; 2023 [cited 2023Dec3]. Available from: https://figshare.com/articles/dataset/_b_PERFIL_DA_PRODU_O_CIENT_FICA_SOBRE_CONSTRU_O_VALIDA_O_E_APLICA_O_DE_TECNOLOGIAS_NA_ENFERMAGEM_estudo_bibliom_trico_b_/23971698/1

11. Oliveira Araújo WC. Recuperação da informação em saúde: construção, modelos e estratégias. ConCI. 2020;3(2):100–34. https://doi.org/10.33467/conci.v3i2.13447

12. Cassiano A do N, Silva CJDA, Nogueira ILA, Elias TMN, Teixeira E, Menezes RMPD. Validação de tecnologias educacionais: estudo bibliométrico em teses e dissertações de enfermagem. Rev. Enferm. Cent.-Oeste Min. 2020;10. https://doi.org/10.19175/recom.v10i0.3900

13. Khabsa M, Elmagarmid A, Ilyas I, Hammady H, Ouzanni M. Learning to identify relevant studies for systematic reviews using random forest and external information. Mach Learn. 2016; 102: 465–82. https://doi.org/10.1007/s10994-015-5535-7.

14. Pagani RN, Pedroso B, dos Santos CB, Picinin CT, Kovaleski JL. Methodi Ordinatio 2.0: revisited under statistical estimation, and presenting FInder and RankIn. Qual Quant. 2023;57(5):4563–602. https://doi.org/10.1007/s11135-022-01562-y

15. Van Eck NJ, Waltman L. Vosviewer Manual. Leiden: Universiteit Leiden; 2020.
