# Supplementary material for: Profile of scientific production on nursing technology construction, validity and application: a bibliometric study
Source: Rev Bras Enferm. 2024 Jul 29;77(3):e20230452. doi: 10.1590/0034-7167-2023-0452 (PMC11290734; doi:10.1590/0034-7167-2023-0452)
Supplement: Translation

# Translation: Perfil da produção científica sobre construção, validação e aplicação de tecnologias em enfermagem: estudo bibliométrico

**DOI:** 10.1590/0034-7167-2023-0452pt

**Authors:** Fernando Conceição de Lima, Taís dos Passos Sagica, João Lucas Moraes Souza, Marta Lenise do Prado, Mary Elizabeth de Santana, Ivonete Vieira Pereira Peixoto, Rubenilson Caldas Valois

**Affiliations:** I Universidade do Estado do Pará. Belém, Pará, Brasil; II Universidade Federal de Santa Catarina. Florianópolis, Santa Catarina, Brasil

**Keywords:** Pesquisa Metodológica em Enfermagem, Enfermagem, Tecnologia, Tecnologia Educacional, Bibliometria

## Abstract

**Objetivo:**

analisar o perfil da produção científica sobre construção, validação e aplicação de tecnologias em enfermagem.

**Métodos:**

trata-se de estudo bibliométrico, realizado em seis bases de dados, fundamentado na aplicação da *Methodi Ordinatio*, disposto em nove etapas. Para representação dos achados, foi utilizado o *software VOSviewer* ^®^.

**Resultados:**

foram identificadas 346 publicações, obtidas nas bases BDENF, CINAHL, EMBASE, LILACS, PubMed/MEDLINE, Scopus e *Web of Science*. Houve predomínio do idioma inglês e 20% dos autores detêm mais de 25% das produções. Apenas duas revistas são responsáveis por 25% das publicações no período pesquisado. Foram selecionadas 26 publicações para a classificação *InOrdinatio*. A temática destaque das publicações foi o Processo de Enfermagem (23%). A tecnologia mais produzida foi *software* (27%) e 50% das obras descrevem construção e validação.

**Conclusões:**

há ênfase na criação de tecnologias educacionais, sobretudo de informática. Os dados exibem oportunidades para futuras pesquisas na área.

## INTRODUÇÃO

O processo de construção de aparatos tecnológicos permeia diversos formatos e moldes. A pesquisa metodológica é o tipo que se volta para o desenvolvimento de produtos e costuma envolver métodos complexos. É uma metodologia que pode ainda ser aplicada às investigações dos formatos de obtenção e organização de dados, além da condução de pesquisas rigorosas^(1-2)^.

Ela é capaz de lidar com o desenvolvimento, a validação e a avaliação de ferramentas e métodos de pesquisa. Nesse tipo de estudo, o pesquisador tem como meta a elaboração de um produto confiável e utilizável que possa ser empregado por outros pesquisadores e outras pessoas. Esse método é aplicável em qualquer área científica, lidando com fenômenos complexos, como o comportamento ou a saúde dos indivíduos^(1-2)^.

No campo da enfermagem, há uma grande quantidade de estudos metodológicos com vistas à produção de tecnologias assistenciais, gerenciais e/ou educacionais. Além dessas, é comum encontrar propostas de avaliação e adaptação cultural de instrumentos internacionais para a utilização no país de interesse^(3-4)^. De modo geral, o enfoque dos estudos metodológicos se volta para o desenvolvimento de construtos aplicáveis na ciência e na profissão de enfermagem^(5)^.

Como característica principal, esses estudos apresentam-se em forma de processos encadeados, iterativos e interativos, nos quais repousam os estágios de desenvolvimento de uma tecnologia. Assumem-se atualmente alguns modelos de divisão, como a realização de diagnóstico situacional, as organizações de revisões de literatura, a construção da tecnologia, a validação e a testagem^(3,6)^.

De modo amplo, o termo “tecnologia” pode se referir a técnicas, métodos, instrumentos, procedimentos e equipamentos que promovem a execução e geram produtos e serviços. Na área da saúde, sobretudo no que se refere à prestação de serviços de enfermagem, a adoção de tecnologias é capaz de refinar as práticas profissionais, as relações interpessoais e o gerenciamento dos processos dentro dos serviços^(2,6)^. Desse modo, a aplicação de tecnologias ocorre de diversas maneiras e tem o seu significado atribuído de acordo com a sua utilização^(3)^.

Diante da visibilidade científica que os estudos metodológicos ganharam e do engajamento da enfermagem na produção desses estudos, questiona-se como esses são utilizados por pesquisadores na elaboração de novas pesquisas^(7)^. Dessa forma, a realização deste estudo propõe uma análise da atividade científica, pelo estudo quantitativo, sobre a utilização das diferentes etapas nas pesquisas metodológicas em enfermagem, uma vez que não há um consenso na literatura sobre o processo metodológico em criar, validar e aplicar tecnologias em enfermagem, assim como explorar métodos e aspectos que contribuam para reflexões sobre a produção e o avanço científico nesse campo^(7-8)^.

## OBJETIVO

Analisar o perfil bibliométrico da produção científica sobre construção, validação e aplicação de tecnologias em enfermagem.

## MÉTODOS

### Aspectos éticos

A pesquisa não foi submetida à apreciação em Comitê de Ética em Pesquisa com Seres Humanos, haja vista que foi realizada com dados secundários e de domínio público. No entanto, preservaram-se os direitos autorais dos estudos.

### Desenho, período e local do estudo

Trata-se de estudo bibliométrico acerca da construção, validação e aplicação de tecnologias em enfermagem. Esse método caracteriza-se por evidenciar métricas, indicadores, produção e disseminação científica de determinado tema^(9)^.

Fundamentou-se na *Methodi Ordinatio*, uma ferramenta multicritério de auxílio que permite ordenar os artigos considerando três fatores: número de citações; Fator de Impacto (métrica); e ano de publicação^(8)^. Além disso, utilizaram-se as diretrizes do fluxograma do *Preferred Reporting Items for Systematic Reviews and Meta-Analyses* (PRISMA)^(10)^, presente no protocolo de estudo registrado na plataforma *Figshare* ^(11)^.

Dessa forma, este estudo seguiu as nove etapas propostas: 1) definição da intenção da pesquisa; 2) pesquisa preliminar nas bases de dados; 3) definição e combinação das palavras-chave e bases de dados; 4) busca definitiva e coleta de dados; 5) procedimento de filtragem; 6) identificação do Fator de Impacto, ano de publicação e número de citações; 7) classificação dos artigos no *InOrdinatio* utilizando a planilha *Rankln*; 8) localização dos textos em formato integral; 9) leitura sistemática e análise dos artigos^(12)^.

Foi realizada a busca preliminar nas seguintes bases de dados: Base de Dados de Enfermagem (BDENF); *Cumulative Index to Nursing and Allied Health* (CINAHL); EMBASE; Literatura Latino-Americana e do Caribe em Ciências da Saúde (LILACS); MEDLINE via *National Library of Medicine* (PubMed); Scopus; e *Web of Science* (WoS). Os dados foram coletados no mês de maio a junho de 2023.

### População ou amostra; critérios de inclusão ou exclusão

Na primeira etapa, utilizou-se a estratégia PICo^(13)^ para formular a questão de pesquisa “Qual o perfil das produções científicas sobre construção, validação e aplicação de tecnologias em enfermagem?”, sendo P (problema): tecnologias em enfermagem, I (interesse): estudos de construção, validação e avaliação, e Co (contexto): enfermagem em seus múltiplos cenários de intervenção.

Foram incluídos artigos científicos sobre a construção, validação e/ou aplicação de tecnologias em enfermagem, disponibilizados em português, espanhol e inglês. Utilizou-se o recorte temporal a partir de 2011, pois nesse ano houve o crescimento de publicações (teses e dissertações) acerca do tema na área, além da expansão dos programas de pós-graduação, em especial, programas profissionais^(8)^. Foram excluídos artigos em formatos de editoriais, cartas ao editor e artigos de opinião.

### Protocolo do estudo

Após a segunda etapa de pesquisa preliminar nas bases de dados, deu-se início à busca dos estudos por meio do acesso remoto ao conteúdo da Comunidade Acadêmica Federada (CAFe), um recurso disponibilizado pela Coordenação de Aperfeiçoamento de Pessoal de Nível Superior (CAPES) e pelo Ministério da Educação (ME), assinado pela Universidade do Estado do Pará (UEPA), por meio da combinação dos Descritores em Ciências da Saúde (DeCS)/*Medical Subject Heading*s (MeSH)/*Emtree* (vinculado ao *Embase Index*), com o auxílio dos operadores booleanos *AND* e *OR*.

Na terceira etapa, para a definição das palavras-chave, utilizaram-se os idiomas português e inglês com a seguinte combinação: (“pesquisa metodológica em enfermagem” *OR* “*nursing methodology research*”) *AND* (“enfermagem” *OR* “*nursing*”) *AND* (“tecnologia educacional” OR “*educational technology*” *OR* “tecnologia” *OR* “*technology*” *OR* “tecnologias” *OR* “*technologies*”). Essa estratégia de busca foi padronizada nas bases de dados e portais eletrônicos, conforme apresentado no Quadro 1.

**Quadro 1:** Estratégia de busca nas bases de dados e portais eletrônicos, 2023

Banco de dados	Estratégia de busca
BDENF/ LILACS	(“pesquisa metodológica em enfermagem” *OR* “*nursing methodology research*”) *AND* (“enfermagem” *OR* “*nursing*”) *AND* (“tecnologia educacional” *OR* “*educational technology*” *OR* “tecnologia” *OR* “*technology*” *OR* “tecnologias” *OR* “*technologies*”) *AND* (db:(“LILACS” *OR* “BDENF”)) *AND* (*year_cluster*: [2011 *TO* 2023])
CINAHL	(“*nursing methodology research*”) *AND* (“*nursing*”) *AND* (“*educational technology*” *OR* “*technology*” *OR* “*technologies*”)
EMBASE	(*‘nursing methodology research’*/exp *OR ‘nursing methodology research’*) *AND* (*‘nursing’*/exp *OR ‘nursing’*) *AND* (*‘educational technology’*/exp *OR ‘educational technology’ OR ‘technology’*/exp *OR ‘technology’ OR ‘technologies’*) *AND* [2011-2023]/py *AND* ([english]/lim *OR* [portuguese]/lim *OR* [spanish]/lim)
MEDLINE/ PubMed	(“*nursing methodology research*”[All Fields]) *AND* (“*nursing*”[*All Fields*]) *AND* (“*educational technology*”[*All Fields*] *OR* “*technology*”[*All Fields*] *OR* “*technologies*”[*All Fields*])) *AND* (2011:2023[pdat])
Scopus	TITLE-ABS-KEY (“*nursing methodology research*”) *AND* (“*nursing*”) *AND* (“*educational technology*” *OR* “*technology*” *OR* “*technologies*”)) *AND PUBYEAR* > 2010 *AND PUBYEAR* < 2024
*Web of Science*	“*nursing methodology research*” (*All Fields*) *AND* “*nursing*” (*All Fields*) *AND* “*educational technology*” *OR* “*technology*” *OR* “*technologies*” (*All Fields*)

Na quarta etapa, busca definitiva e coleta de dados, os resultados foram exportados no formato *Research Information Systems* (RIS) para o *software* gerenciador de revisões Rayyan^®(14)^. Na quinta etapa, foram detectados e excluídos os documentos duplicados, e os demais artigos foram analisados por meio da leitura dos títulos e resumos por três pesquisadores, sendo um especialista e dois doutores, de maneira independente, responsáveis pela seleção em duas etapas, com mascaramento, na perspectiva de manter o rigor metodológico e a revisão dupla-cega.

Os conflitos entre eles foram decididos por um terceiro pesquisador, com título de doutor, acionado para definir a seleção. Foram utilizados os programas JabRef^®^ e o *software Microsoft Excel* ^®^ para a construção do portfólio bibliográfico. Na sexta etapa, houve a identificação do Fator de Impacto, ano de publicação e número de citações pelo *Google Scholar* ^®^. Na sétima etapa, houve a geração do valor *InOrdinatio* de cada artigo utilizando a planilha *Rankln*, versão 2.0. Esse valor foi adquirido por meio da fórmula *InOrdinatio* 2.0 = {(∆* IF) - [λ* (*ResearchYear - PubYear*)/*HalfLlife*] + Ω* ∑ Ci/[(*ResearchYear*+1) -*PubYear*]}^(15)^. Foi estabelecido 10 (maior importância) para as constantes ∆ (Fator de Impacto), λ (ano de publicação), Ω (número de citações). Dessa forma, os artigos foram classificados de forma decrescente conforme o resultado obtido.

A oitava etapa foi realizada concomitantemente à sexta etapa, na qual foi realizado o *download* dos artigos selecionados. A nona etapa foi atendida parcialmente, por ter sido realizada apenas a leitura flutuante dos artigos selecionados para compor o portifólio bibliográfico desta revisão, visto que a leitura na íntegra dos estudos não é o objetivo da pesquisa bibliométrica.

### Análise dos resultados e estatística

Para a análise dos resultados, foram aplicadas as três leis clássicas da bibliometria: I) Lei de Lotka: para analisar a produtividade dos autores; II) Lei de Bradford: visando avaliar a produtividade dos periódicos; III) Lei de Zipf: para avaliar a frequência de palavras-chave^(8)^.

A organização dos dados se deu em planilhas do *Microsoft Excel* ^®^. Os dados foram analisados descritivamente e, para a representação dos achados das Leis de Lokta e Zipf, utilizou-se o *software Visualizing Scientific Landscapes* (*VOSviewer* ^®^), versão 1.6.19, que consiste em uma representação referente à ocorrência dos termos, bem como suas relações, na qual a espessura das conexões indica a intensidade da cooperação entre os termos^(16)^.

## RESULTADOS

A busca inicial, conforme os critérios de inclusão, resultou em um total de 346 publicações, sendo essas oriundas das seguintes bases de dados: BDENF (25); CINAHL (7); EMBASE (1); LILACS (25); PubMed/MEDLINE (159); Scopus (124); e *Web of Science* (5). No *software* Rayyan^®^, foram identificados e excluídos 124 artigos duplicados, restando o quantitativo de 222 estudos, bem como as informações sobre os principais periódicos de publicação, autores e idioma de origem; esses fizeram parte do escopo para serem analisados segundo as Leis de Lotka, Bradford e Zipf. Após a leitura do título e do resumo dos 222 artigos no *software* Rayyan^®^, 196 foram excluídos por não estarem intimamente ligados ao tema. Dessa forma, a seleção final dos estudos para compor o portifólio bibliográfico foi de 26 artigos e serem analisados pelo *Methodi Ordinatio*.

Destaca-se que as publicações se distribuíram em 76 periódicos, sobressaindo-se as revistas *Nurse Educator* (42; 19%), *Computers, Informatics, Nursing* (15; 6,8%), *Nurse Education in Practice* (10; 4,51%), *Journal of Nursing Education* (9; 4%), *Nurse Education Today* (9; 4%), Revista Brasileira de Enfermagem (8; 3,6%), *Midwifery* (6; 2,7%), *Journal of Advanced Nursing* (5; 2,25%), *Nurse Researcher* (5; 2,25%) e *Practising Midwife* (5; 2,25%). Juntas somam 114 publicações, correspondendo a aproximadamente 51,23% das publicações. O detalhamento dessa distribuição encontra-se na Tabela 1, na qual os periódicos descritos representam os quadrantes 1 e 2.

**Tabela 1:** Produção científica sobre construção, validação e aplicação de tecnologias em enfermagem segundo as Leis de Bradford, Lokta e Zipf, 2011-2023

Lei de Bradford			
**Quadrante**	**Revistas**	**Produção**	**%**
1	2	57	25,68
2	8	57	25,68
3	20	54	24,32
4	46	54	24,32
Total	76	222	100
**Lei de Lokta**			
**N° de autores**	**% de autores**	**N° de produções**	**% de produções**
130	20	178	25,39
571	80	523	74,61
Total	100	701	100
**Lei de Zipf**			
**Zona**	**Total de palavras**	**Frequência**	**%**
Trivial	22	1482	41,89
Interesse	192	1225	34,63
Ruido	697	830	23,48
Total	911	3537	100

O quantitativo total de autores foi de 653, responsáveis por 701 autorias, sendo que o número de documentos de acordo com a quantidade de autores se deu da seguinte forma: um autor (Charalambous, A) publicou quatro artigos; dois autores publicaram três artigos; 41 autores publicaram dois artigos; e 609 autores publicaram apenas um artigo. Nesse sentido, 20% dos autores foram responsáveis por 25,39% das produções, conforme explicitado na Tabela 1.

Quanto à linguagem das publicações, houve o predomínio de publicações na língua inglesa (208; 93,69%), seguida da portuguesa (12; 5,41%), alemã (1; 0,45%) e espanhola (1; 0,45%). No que se refere ao quantitativo de artigos publicados por ano, tem-se os seguintes quantitativos: 2011 (27 - 12,16%); 2012 (43 - 19,37%); 2013 (31 - 13,96%); 2014 (26 - 11,71%); 2015 (16 - 7,21%); 2016 (15 - 6,76%); 2017 (14 - 6,31%); 2018 (14 - 6,31%); 2019 (16 - 7,21%); 2020 (9 - 4,05%); 2021 (5 - 2,25%); 2022 (4 - 1,80%); e 2023 (2 - 0,90%). O ano de 2012 destacou-se com o maior quantitativo.

A análise da rede de coautoria está exposta na Figura 1. Com o emprego do filtro de pelo menos duas ocorrências de autores nos artigos, foram gerados 14 *clusters* e um total de 642 itens, com 23 conexões entre os autores. Observa-se a influência científica de determinados autores, responsáveis pela concentração do foco de produções científicas e por maiores números de conexões com os demais.

**Figura 1:** Rede de coautoria da produção científica sobre construção, validação e aplicação de tecnologias em enfermagem. 2011-2023

Com relação às palavras-chave, foram encontrados 911 termos, com uma frequência geral de 3.537 repetições, sendo categorizadas em três zonas, como informação trivial (22), informação interessante (192) e ruído (697), conforme o número de repetições. O detalhamento do resultado da Lei de Zipf encontra-se na Tabela 1.

A análise da distribuição de palavras-chave foi efetuada por meio do *software VOSviewer* ^®^ e está representada na Figura 2. Nessa avaliação, foi utilizado o filtro de, no mínimo, duas ocorrências da palavra nos artigos, ocasionando a aparição de 351 *clusters*, dos quais foram selecionados 26 para compor esta revisão. Na Figura 2, nota-se a disposição dos termos nos idiomas português e inglês, destacando-se a palavra-chave *nursing methodology research*, com 179 ocorrências nos textos.

**Figura 2:** Rede de distribuição das palavras-chave da produção científica sobre construção, validação e aplicação de tecnologias em enfermagem. 2011-2023

O Quadro 2 caracteriza o perfil das 26 publicações selecionadas classificadas conforme o *InOrdinatio*, mediante Fator de Impacto (FI) do periódico, número de citações (CI) e ano de publicação. Apenas três estudos (1, 2 e 3) possuem *InOrdinatio* maior que 80, ou seja, um melhor portifólio bibliográfico do estado da arte, e, dos 26 estudos, somente dois possuem *InOrdinatio* menor que um.

**Quadro 2:** Caracterização das da produção científica sobre construção, validação e aplicação de tecnologias em enfermagem segundo o *InOrdinatio.* 2011-2023

ID	Título	Autores	Periódico	Ano de publicação	CI	FI	*InOrdinatio*
1^(17)^	*Evaluation of vSim for Nursing in an Adult Health Nursing Course: A Multisite Pilot Study.*	Wright RR *et al*.	*CIN: Computers, Informatics, Nursing*	2018	43	2,1	86,08772
2^(18)^	*Validation of an educative manual for patients with head and neck cancer submitted to radiation therapy*	Cruz FOAM *et al*.	Revista Latino-Americana de Enfermagem	2016	75	-	84,53947
3 ^(19)^	*Validation of educational material for the care of people with intestinal stoma*	Sena JF *et al*.	Revista Latino-Americana de Enfermagem	2020	34	-	81,05263
4 ^(20)^	*Mobile health technology for gestational care: evaluation of the GestAção’s app*	Silva RM *et al*.	Revista Brasileira de Enfermagem	2019	30	1,3	67,73684
5 ^(21)^	Jogo educativo de administração de medicamentos: um estudo de validação	Moreira APA *et al*.	Revista Brasileira de Enfermagem	2014	54	1,3	55,15789
6 ^(22)^	Construção de hipermídia para apoio ao ensino da sistematização da assistência de enfermagem	Salvador PTCO *et al*.	Revista Gaúcha de Enfermagem	2019	23	1,1	51,73684
7 ^(23)^	*Educational hypermedia in nursing assistance at birth: building and validation of content and appearance*	Oliveira LL *et al*.	Revista Brasileira de Enfermagem	2019	21	1,3	49,73684
8 ^(24)^	*Assessment of a prototype for the Systemization of Nursing Care on a mobile device*	Rezende LCM *et al*.	Revista Latino-Americana de Enfermagem	2016	39	-	39,53947
9 ^(25)^	*Structuring methodology of the Computerized Nursing Process in Emergency Care Units*	Paese F *et al*.	Revista Brasileira de Enfermagem	2018	18	1,3	36,42105
10 ^(26)^	Desenvolvimento de Ambiente Virtual de Aprendizagem em Enfermagem sobre ressuscitação cardiorrespiratória em neonatologia	Rodrigues RCV *et al*.	Revista da Escola de Enfermagem da USP	2013	42	1,1	36,02392
11 ^(27)^	*Validation of an educational manual for breast cancer patients undergoing radiotherapy*	Cruz FOAM *et al*.	Revista Latino-Americana de Enfermagem	2020	15	-	33,55263
12 ^(28)^	*Plataforma PEnsinar^®^: a learning tool for teaching the nursing process*	Melo ECA *et al*.	Revista Brasileira de Enfermagem	2018	16	1,3	33,08772
13 ^(29)^	Construção e validação de uma tecnologia educacional para prevenção da sífilis congênita	Costa CC *et al*.	Acta Paulista de Enfermagem	2020	10	1,1	32,05263
14 ^(30)^	*Software for systematization of nursing care in medical units*	Silva Junior MG *et al*.	Revista Brasileira de Enfermagem	2018	14	1,3	29,75439
15 ^(31)^	Construção e validação de videoaula sobre ressuscitação cardiopulmonar	Alves MG *et al*.	Revista Gaúcha de Enfermagem	2019	12	1,1	29,73684
16 ^(32)^	*T-NDX Diagram: Educational Technology Used to Teach Diagnostic Reasoning Based on Nursing Theories.*	Lopes ROPR *et al*.	*International Journal of Nursing Knowledge*	2020	4	2,1	27,05263
17 ^(33)^	*Soulage-tavie: Development and validation of a virtual nursing intervention to promote self-management of postoperative pain after cardiac Surgery*	Martorella G, Côté J, Choinière M	CIN: *Computers, Informatics, Nursing*	2013	16	2,1	22,38756
18 ^(34)^	*Validation of a booklet for the correct use of personal protective equipment in the context of COVID-19*	Silva ABP *et al*.	Texto & Contexto Enfermagem	2021	3	1,4	21,36842
19 ^(35)^	*Virtual Guide On Ocular Self-Examination To Support The Self-Care Practice For People With HIV/AIDS*	Lima MA *et al*.	Revista da Escola de Enfermagem da USP	2014	19	1,1	18,15789
20 ^(36)^	*Development of a mobile application focusing on developmental support care for Korean infants born prematurely: a methodological study.*	Park JH, Cho H	*Child Health Nursing Research*	2022	1	0,9	12,68421
21 ^(37)^	*Construction of a nursing care protocol for children in post-hematopoietic stem cell transplantation*	Rodrigues J *et al*.	Revista Gaúcha de Enfermagem	2022	0	1,1	9,684211
22 ^(38)^	*A clinical nurse specialist intervention to facilitate safe transfer from ICU*	St-Louis L, Brault D	*Clinical Nurse Specialist* (CNS)	2011	33	-	9,595142
23 ^(3)^	Tecnologia educacional para pessoas com doença renal crônica: construção e validação de conteúdo	Santos FGT *et al*.	Revista de Pesquisa	2021	3	-	7,368421
24 ^(39)^	Análise de qualidade de objeto virtual de aprendizagem para avaliação da dor em enfermagem	Alvarez AG *et al*.	*Revista Cubana de Enfermería*	2018	4	0,3	3,087719
25 ^(40)^	Construção e avaliação de um ambiente virtual de aprendizagem para liga de segurança do paciente	Moraes AIS *et al*.	CuidArte Enfermagem	2021	1	-	0,701754
26 ^(41)^	Avaliação de objeto virtual de aprendizagem sobre raciocínio diagnóstico: um estudo descritivo	Costa CPV *et al*.	*Online Brazilian Journal of Nursing*	2015	5	0,1	-3,97076

*Legenda: ID - identificação; FI - Fator de Impacto do periódico; CI - número de citações.*

A temática de destaque das publicações foi o Processo de Enfermagem (PE) (6; 23%), seguido de fundamentos de enfermagem (12%) e infectologia (12%), com três publicações cada. As temáticas enfermagem cirúrgica (8%), neonatologia (8%), obstetrícia (8%) e oncologia (8%) tiveram duas publicações cada. Os temas com uma publicação foram hematologia (4%), nefrologia (4%), saúde do adulto (4%), segurança do paciente (4%), teorias de enfermagem (4%) e urgência e emergência (4%).

Foram descritos nas publicações os seguintes artefatos tecnológicos: *software* (7; 27%), ambiente virtual (6; 23%), cartilha (5; 19%), hipermídia (2; 8%), manual (2; 8%), instrumento (1; 4%), jogo (1; 4%), protocolo (1; 4%) e videoaula (1; 4%). No que se refere às etapas descritas, 13 publicações (50%) apresentaram construção e validação das tecnologias, oito (31%) detalharam a construção, três (12%) apresentaram aplicação e duas (8%) apresentaram validação.

## DISCUSSÃO

Há menos de dez anos, a inovação e o desenvolvimento tecnológico eram uma prática científica ainda incipiente no campo da enfermagem. No entanto, é sabido que a proposição e o uso de tecnologias possibilitam melhorias na qualidade da assistência em saúde, com um melhor custo efetivo e eficiência, já que as redes de conhecimento são utilizadas para a construção de produtos advindos de sua práxis e que propiciam a inovação tecnológica para se aproxima da realidade dos usuários^(5)^.

Por outro lado, os estudos metodológicos estreiam na enfermagem no ano de 2006 e apresentam um aumento considerável em publicações a partir do ano de 2015^(42)^, tornando-se imperativo desenvolver as tecnologias em suas diferentes etapas, tais como criação, validação e aplicação de produtos e processos para diferentes fins^(6)^.

Ressalta-se que a maior porcentagem de produções presentes neste estudo corresponde a pesquisas publicadas no idioma inglês e com crescimento exponencial ao longo das duas últimas décadas, sendo a maior parte dos estudos publicados em 2012. O desenvolvimento tecnológico passou a exercer forte impacto na produção do conhecimento científico, na enfermagem e na saúde, tendo em vista as múltiplas demandas individuais e coletivas que se apresentam na assistência e na educação em saúde^(43)^.

Além disso, tem-se o inglês como uma língua universal também para a ciência, já que possibilita que todos os pesquisadores em todo o mundo tenham a possibilidade de se comunicar e trocar informações utilizando um único idioma. Como consequência desse ato, há um aumento de publicações em inglês ao longo dos anos como reflexo dos esforços de cientistas, instituições de ensino e periódicos na perspectiva de internacionalizar os estudos científicos^(8)^.

Como resultado dos estudos avaliados pelo método *InOrdinatio*, tem-se que a maioria dos estudos é produto de autores brasileiros, mas também possui tradução e divulgação de seus textos para o idioma inglês, o que reforça a estratégia de utilização do inglês como uma medida de internacionalização das produções científicas brasileiras, além do aumento do Fator de Impacto, maior possibilidade de leitura e citação quando se oportuniza um maior acesso aos estudos^(44)^.

A ampliação de acesso às publicações está diretamente relacionada a uma maior qualificação e prestígio dos periódicos para que a produção da enfermagem avance em qualidade e visibilidade. Periódicos de prestígio buscam veículos consagrados, como importantes bases de dados, bibliotecas e repositórios científicos, para a divulgação de suas produções científicas, na perspectiva de garantir reconhecimento e interlocução entre a comunidade científica em diferentes campos de interesse^(45)^.

Além disso, os resultados desta revisão apontam para uma concentração de autoria, haja vista que um autor publicou quatro artigos e 609 autores publicaram apenas um artigo cada. Esse achado corrobora a Lei de Lotka, que descreve o padrão da produção científica dos autores, ou seja, afirma que poucos autores, autoridades em um assunto, produzem muito, e muitos autores, provavelmente com menor prestígio e amplo espectro de estudo, produzem pouco sobre um mesmo tema. Essa lei permite verificar a produtividade dos autores e os centros de pesquisa mais desenvolvidos em determinada área^(46-47)^.

Estudos que permitem identificar a produtividade e centros de excelência em determinada área são importantes para a consolidação e fortalecimento desse campo de investigação. Essa perspectiva é ressaltada em estudo documental que chama a atenção para a adoção e condução de linhas de pesquisa na enfermagem que envolvam e apresentam temas semelhantes em pesquisas científicas com tradições investigativas, produzem projetos com afinidades entre si e com um maior direcionamento da produção, contribuindo para a sistematização do conhecimento científico^(48)^.

Sobre a Lei de Zipf, mostrou-se que um menor número de palavras foi citado muitas vezes e que muitas palavras foram citadas poucas vezes, portanto, um resultado inversamente proporcional. Como esperado, a palavra “*nursing methodoly research*” teve maior ocorrência, seguida de elementos que são componentes, como “*nursing research education*” e “*learning*”, uma vez que o estudo objetiva analisar o perfil bibliométrico da produção científica sobre construção, validação e aplicação de tecnologias em enfermagem.

Os estudos metodológicos possibilitam a investigação para obtenção e organização dos dados para construir, validar e avaliar, segundo preceitos metodológicos rigorosos, como métodos de pesquisa, para elaboração de instrumentos confiáveis e que possuam aplicabilidade em diferentes realidades e em diferentes áreas do conhecimento, como na enfermagem, na perspectiva de criar e validar diferentes produtos tecnológicos, a exemplo das tecnologias educacionais (TE)^(49)^.

O tipo de estudo metodológico foi o mais abordado nas pesquisas encontradas, oriundos principalmente de estudos de construção e validação. A primeira fase dos estudos metodológicos (produção) é construída com base em evidências científicas presentes na literatura, procedendo com a revisão dos estudos disponíveis na literatura e evidência de realidade, que necessita de estudos primários tanto para o diagnóstico situacional quanto para promover a construção compartilhada e propiciar a construção da tecnologia^(50)^.

Em relação às áreas do conhecimento, identificou-se que a aplicação das tecnologias esteve mais relacionada ao PE. O uso de tecnologias propicia a adesão ao PE pelos enfermeiros quando comparados a índices antes da utilização de tecnologias^(51)^. Além disso, evidencia-se o interesse da enfermagem pela execução de um cuidado sistematizado e de qualidade, assim como pelo rigor em cumprir a Resolução nº. 358 de 2009 do Conselho Federal de Enfermagem (COFEN) em tornar obrigatório o uso do PE nos serviços de saúde públicos ou privados^(52)^.

Tecnologias, quando desenvolvidas para o uso educacional, são dotadas de conhecimentos que visam produzir/preparar, aplicar/intervir e acompanhar um processo educativo. Além disso, são instrumentos que possibilitam a avaliação da educação em saúde, pois viabilizam ações de saúde, apoiadas por TE, que se aproximam da realidade dos indivíduos e promovem uma maior qualidade de vida^(50)^. O ato de criar uma TE, com base em evidências científicas, permite que o público-alvo tenha acesso a um conglomerado de conteúdos disponibilizados de forma fácil, prático e atual, com a disponibilização de informações seguras em relação ao conteúdo abordado^(53)^.

Tem-se que a produção de uma tecnologia surge a partir da percepção da necessidade entendida na discussão, compartilhamento de saberes e pelo estímulo à criatividade^(54)^. Isso reflete no aumento de produções relacionadas à construção e validação de TE, já que constituem benefícios para a população com expressiva melhoria na qualidade de vida, promovendo a adoção de comportamentos saudáveis, transformação de práticas, inovação no modo de aprender a aprender e por fortalecer a aprendizagem significativa com autonomia e empoderamento^(55-56)^.

A etapa de validação está relacionada a diferentes aspectos, como a validação de um conteúdo, aparência, semântica, usabilidade, aplicabilidade, ludicidade, interatividade, entre outras ações. Considera-se que há a possibilidade de diferentes interações no momento de validação, o que exige planejamento para o desenvolvimento das etapas, podendo ser concomitante ou sequencial^(6)^. Tornar válida uma tecnologia com enfoque educacional é formalizar a relevância de um conteúdo desenvolvido na perspectiva de englobar informações que sejam adequadas e seguras para uso de maneira educativa, seguindo os preceitos metodológicos exigidos^(57)^.

Dessa forma, é importante que, após a criação de uma TE, o próximo passo seja a validação; essa etapa é realizada por especialistas com *expertise* na área de estudo apresentada. O Ministério da Saúde (MS) objetiva que a produção de tecnologias possa conter uma linguagem clara, acessível, inteligível e adequada à realidade do público-alvo, já que pode ajudar ou atrapalhar a transmissão da mensagem desejada; logo, a experiência de especialistas é fundamental para que a TE seja considerada válida^(58)^.

No que diz respeito ao artefato tecnológico produzido, houve a prevalência de *softwares*, que são um conjunto lógico de informações que utiliza algoritmos para o processamento dos dados e que resultam em um tipo de programa^(59)^. No contexto educacional, esses artefatos promovem novas experiências de aprendizagem e, quando utilizados para compor um material educacional, evidenciam uma prática inovadora no processo de ensino e aprendizagem^(60)^.

### Limitações do estudo

A montagem do *string* de busca representou uma etapa crítica, uma vez que a escolha inadequada de descritores pode resultar na exclusão de informações relevantes. Além disso, o número de publicações associadas aos *strings* de busca pode ser uma limitação, pois um número pouco expressivo pode indicar uma pesquisa muito restritiva. Essas limitações tornaram o desenvolvimento e a abordagem cuidadosos e criteriosos na condução da pesquisa, visando mitigar esses obstáculos e garantir a integridade e a relevância dos resultados.

### Contribuições para as áreas da enfermagem, saúde ou políticas públicas

O presente estudo explora o que tem sido produzido pela área da enfermagem no que tange ao desenvolvimento de tecnologias, sobretudo aquelas embasadas em métodos científicos consolidados e bem descritos na literatura. Portanto, esta pesquisa carrega consigo dados que podem subsidiar novos estudos metodológicos, além de trazer *insights* que possibilitam a formação de redes colaborativas para produção de tecnologias.

## CONCLUSÕES

O desenvolvimento de tecnologias tem um papel fundamental na consolidação da enfermagem enquanto ciência. É por meio do processo de construção e validação daquilo que se cria para a profissão que a autonomia da classe se evidencia. Neste estudo, evoca-se uma variedade de estratégias adotadas pelos autores dos trabalhos analisados na busca do desenvolvimento de suas tecnologias. Isso fortalece a característica de pluralidade presente na temática das produções tecnológicas: a infinidade de possibilidades a serem desenvolvidas.

Há ainda um forte apelo para a produção de TE. A tendência em desenvolver produtos voltados para o ensino em enfermagem, sobretudo relacionados às tecnologias da informação (*softwares*, aplicativos e suporte de informática), é característica mundial e se apresenta nos diversos contextos de atuação da profissão, como a prática clínica, o gerenciamento do cuidado e a melhoria de processos gerais institucionais.

Com os dados deste perfil, destaca-se ainda o potencial para estudos futuros, uma vez que a apreciação do tema de produção bibliométrica leva a *insights* capazes de elucidar como a produção tecnológica na enfermagem se dá. Recomenda-se que outras pesquisas sejam realizadas, considerando especificar ainda mais os ramos pelos quais a produção de tecnologia na área da enfermagem pode permear, explorando seus tipos-classificações e outros aspectos.

## Data Availability

O protocolo deste estudo foi registrado na plataforma *Figshare* (https://figshare.com/s/4cc25bdcc1711a586f64).
